# The Structure and Species Co-Occurrence Networks of Soil Denitrifying Bacterial Communities Differ Between A Coniferous and A Broadleaved Forests

**DOI:** 10.3390/microorganisms7090361

**Published:** 2019-09-18

**Authors:** Jie Chen, Jiajia Li, Weijun Shen, Han Xu, Yide Li, Tushou Luo

**Affiliations:** 1 Research Institute of Tropical Forestry, Chinese Academy of Forestry, Longdong, Guangzhou 510520, China; ywfj@163.com (H.X.); liyide@caf.ac.cn (Y.L.); luots@126.com (T.L.); 2 College of Tourism and Planning, Pingdingshan University, Pingdingshan 467000, China; 3 Key Laboratory of Vegetation Restoration and Management of Degraded Ecosystems, South China Botanical Garden, Chinese Academy of Sciences, 723 Xingke Rd. Tianhe District, Guangzhou 510650, China; shenweij@scbg.ac.cn

**Keywords:** Soil N cycling, functional gene, microbial network, artificial plantation, forest degradation

## Abstract

*Acacia mangium* (AM) and *Pinus massoniana* (PM) are widely planted in tropical regions, whereas their effects on soil microbial communities remain unclear. We did a comprehensive investigation of soil denitrifying bacterial communities in AM and PM monoculture plantations in Southern China based on the high throughput sequencing data of their functional genes: *nirK*, *nirS*, and *nosZ*. The average abundance of *nosZ* (1.3 × 10^7^) was significantly higher than *nirS* (5.6 × 10^6^) and *nirK* (4.9 × 10^5^). Shannon estimator revealed a markedly higher α-diversity of *nirS* and *nosZ* communities in PM than in AM plantations. The AM and PM plantations were dominated by different *nirS* and *nosZ* taxa belonging to proteobacteria, actinobacteria, thermoleophilia, chloroflexia, and acidobacteria, while the dominant *nirK* taxa were mainly categorized into proteobacteria in both types of plantations. The structure of *nirS* and *nosZ* communities shifted substantially from AM to PM plantations with changes in soil moisture, NH_4_^+^, and microbial biomass nitrogen content. The species co-occurrence network of *nirK* community was better organized in a more modular manner compared to *nirS* and *nosZ* communities, and the network keystone species mostly occurred in PM plantations. These results indicated a highly species corporation of *nirK* community in response to environmental changes, especially in PM plantations. AM and PM plantations can form different soil denitrifying microbial communities via altering soil physicochemical properties, which may further affect soil N transformations.

## 1. Introduction

A large part of the natural forest has been transformed into different monoculture plantations in the tropical and subtropical regions in the past decades [1,2]. However, the ecological effects of these plantations remain debatable. These forest managements often lead to substantial losses in forest productivity, biodiversity, and soil nutrient stocks [3,4,5,6]. Changes in forest environments are likely to be paralleled with shifts in the soil microbial communities that has potential feedbacks on the ecosystem function and ecological significance [7,8]. A variety of studies have demonstrated that tree species could affect the composition and abundance of soil microorganisms, mainly through altering substrate quality and modifying microhabitats [9,10]. Thus, establishing various types of plantations in the same region may form different soil microbial communities as a reflection of alterations in the soil environments. However, the majority of studies only focused on ubiquitous microorganisms [7,9,11], and the effects of different types of plantations on specific functional microorganisms responsible for N cycling are unclear.

Given the critical role of denitrifying microorganisms in the denitrification processes and gaseous N production, the abundance, diversity, and composition of denitrifier communities are proved to be related to soil N_2_O emission in different environments [12,13,14]. N_2_O is a potent greenhouse gas that could exert the greenhouse effect 310 times more than CO_2_ and 12 times stronger than CH_4_ [15]. Thus, the denitrifying microorganisms involved in N_2_O emission under environmental changes merit scientific attention. Tropical and subtropical forest soils are the most abundant natural sources of N_2_O, and an alarming increase of N_2_O emission has been observed in these forests, caused by anthropogenic activities [16]. It was demonstrated that conversion of tropical forests to pastures, agriculture, and economic plantations generally altered the soil N_2_O emission rate [17,18,19,20]. However, the response of denitrifying microorganisms to these land use changes are not well studied. Clarifying the composition and abundance of denitrifying microbial communities in different plantations could help us explore the microbial mechanisms driving the N_2_O emission alternations.

Denitrifiers that harbor nitrite reductase genes (*nirK* and *nirS*) and the N_2_O reductase gene (*nosZ*) are the key drivers of soil N_2_O emission, as they conduct the processes of N_2_O production and consumption [21]. The *nirK* and *nirS* are functionally equivalent but phylogenetically separated genes, which generally do not appear in the same microorganism [21]. *nirK* is a copper-containing gene and *nirS* is a cytochrome *cd1* containing gene. Both of the *nirK* and *nirS* containing microbial communities are widely distributed in the bacterial and archaeal taxa [22,23,24]. Nevertheless, the current denitrifying genes primer sets exhibit amplification biases that preferentially target the bacterial taxa to the exclusion of sequences found in Archaeal denitrifiers [21]. The bacterial and archaeal denitrifiers generally predominate in different ecosystems [25,26]. Archaea are documented as the predominant microbial populations in extreme environments, such as highly saline water and hot springs, where they may sustain the N cycling [27]. Thus, in forest soils, the bacterial denitrifying communities have been widely investigated [25,26,28]. It has been suggested that the *nirK* and *nirS* denitrifying groups respond differently to changes in soil conditions, such as soil moisture [28], pH [29], soil carbon (C), and N contents [25,30,31]. Graf et al. [32] proposed a higher frequent co-occurrence of *nirS* and *nosZ* genes than that of *nirK* and *nosZ* genes, suggesting that the complete denitrification (NO_3_^−^ to N_2_) would likely occur in the *nirS* dominated environments. Although distributions of *nirK*, *nirS*, and *nosZ* bacterial communities and their associated soil properties have been studied in natural forest soils [26,33,34], the features and determinants of these denitrifiers in soils from artificial plantations are elusive. 

Soil microbial communities usually form complex co-occurrence networks via positive or negative interconnections among different taxa, and the structure of networks is closely related to environment conditions [35,36]. Hence, clarifying the properties of networks under various environments can offer a better understanding of microbial responses to environmental changes. Furthermore, network analysis of species co-occurrence patterns provides new insights into keystone species in microbial communities, as well as their responses to habitat conditions [37,38], which can help us to reveal the key drivers for microbial structure shifts. While recent studies have investigated soil microbial networks in response to land use change [39], elevated CO_2_ [35], deforestation [40], precipitation changes [41], among others, the effects of monoculture plantations on these networks is unknown. Most of the previous microbial network studies are focused on soil bacterial and fungal communities, but the characteristics of denitrifying microbial networks in different habitats are unclear.

The *Acacia mangium* and *Pinus massoniana* are increasingly planted as monoculture plantations in cleared forests in tropical and subtropical areas, which may affect soil microbial communities. In this study, we investigated the abundance and structure of the denitrifying bacteria in the *Acacia mangium* and *Pinus massoniana* monoculture plantations in Southern China by quantifying and sequencing the marker genes. A species co-occurrence network was constructed for each denitrifier community, aiming to clearly dissect the species interconnections within the structure and further identify the keystone species [42]. We performed these analyses to (1) compare the abundance, diversity, and structure of the *nirK*, *nirS*, and *nosZ* denitrifying bacteria communities between the *Acacia mangium* and *Pinus massoniana* monoculture plantations and (2) investigate the main influencing factors of the three denitrifying bacterial communities in each type of plantations.

## 2. Materials and Methods 

### 2.1. Study Site

This study was conducted at the Heshan national field research station of forest ecosystem (112°50’E, 22°34´N), Guangdong Province, Southern China. The climate of this region is the typical subtropical monsoon climate, with a mean annual precipitation of 1669 mm and mean annual temperature of 22.5 °C. The soil is classified as laterite (or Oxisols based on the USDA soil taxonomy), and the climax vegetation is evergreen broad-leaved forest (EBLF). However, the natural forest was degraded due to intensive anthropogenic activities. To investigate the effects of different management strategies on the recovery of degraded forests, five coniferous monoculture (*Pinus massoniana*, PM) plantations and five broadleaf monoculture (*Acacia mangium*, AM) plantations were randomly established in a 50-ha cleared forest area in 2005. Each plantation had a total area of approximately 1 ha, and the distance between two plantations was no less than 100 m. Trees were spaced 2 × 3 m, and the tree density was ~ 1650 ha^−1^. 

### 2.2. Soil Sampling and Analyses 

In each plantation, fifteen sampling sites were selected in the open areas between trees to avoid the collection of rhizosphere soils. The distance between any two sampling sites was no less than 20 m, and the fifteen sampling sites were randomly spread in the whole plantation to ensure considerable coverage of the plantation area. At each sampling site, one topsoil (0–10 cm) was collected using an anger (Φ 5 cm). Afterwards, we thoroughly mixed the fifteen samples from one plantation by hand with sterile gloves to form one composite sample. Finally, a total of 10 composite samples were obtained, with 5 in the PM plantations and the other 5 from the AM plantations. After removing root, litter, and stones, soil samples were sieved through a 2 mm mesh and divided into two parts. One part was stored at 4 °C and used for soil physicochemical analyses, and the other part was stored at −20 °C and used for the microbial analyses. All analyses were conducted within two weeks. Soil water content (SWC) was measured through drying fresh soil in an oven at 105 °C to constant weight. Soil pH was tested using a pH meter (Denver Instrument UB-7 pH/ev Meter, USA) in a soil suspension with soil to water ratio of 1:2.5. Soil chemical properties were measured using the methods summarized by Liu et al. [43]. Briefly, soil NH_4_^+^ and NO_3_^−^ were extracted by 2 M KCl, and the contents of NH_4_^+^ and NO_3_^−^ were measured using the indophenol blue colorimetry and copperized cadmium reduction methods, respectively. Soil organic matter (SOM) was determined using the K_2_Cr_2_O_7_ oxidation approach. Soil microbial biomass carbon (MBC) and microbial biomass nitrogen (MBN) were detected through the fumigation method. Generally, two sets of 10 g fresh soil were weighed into glass beakers. One sample was fumigated with chloroform in vacuum glass dryers for 24 h in the dark, then extracted with 0.5 M K_2_SO_4_. The other sample was directly extracted with 0.5 M K_2_SO_4_ without fumigation. The extracted liquid from both the fumigated and non-fumigated soil was loaded on a total organic C analysis instrument (TOC-VCSH, Shimadzu, Japan) to measure the C content. The C content in the non-fumigated soil represented soil dissolved organic carbon (DOC) content, and the difference of C content between fumigation and non-fumigation multiplied by 0.45 was the MBC content. The MBN content was calculated as the difference of N concentration between the fumigation and non-fumigation multiplied by 0.54.

### 2.3. Soil DNA Extraction and Quantification of nirK, nirS, and nosZ Genes

Soil total DNA was extracted and purified with the HiPure Soil DNA Mini Kit (Magen, Guangzhou, China) from 0.3 g fresh soil according to the manufacturer’s instructions. The obtained DNA solution was quantified using a NanoDrop 2000 spectrophotometer (Thermo Fisher Scientific Inc., Wilmington, Delaware, USA). 

The abundance of the *nirK*, *nirS*, and *nosZ* gene was detected using real-time PCR on an ABI 7500 thermocycler system (Applied Biosystems, Foster City, CA, USA). The reaction solution was added in a 96-well plate, including 12.5 μL SYBR Premix Ex Taq (TaKaRa Biotechnology, Tokyo, Japan), 1 μL primer (10 mmol/L), and 2 μL DNA template (1–10 ng). A no template control was conducted by replacing the DNA template with RNase free ultra-pure water. The primers and PCR profiles are presented in Table S1. Three standard curves were constructed during the real-time PCR for the calculation of the final abundance of *nirK*, *nirS,* and *nosZ* genes. The plasmids extracted from clones that contain one of the target genes (*nirK*, *nirS,* and *nosZ*) were diluted to generate a series of standard DNA templates with different concentration (10^3^–10^8^ copies per µL). Then, real-time PCR was conducted with the standard DNA templates, and a standard curve was generated. Gene copy numbers of the soil samples were directly calculated according to the standard curve. The PCR efficiency and correlation coefficients (R^2^) for standard curves were ≥ 90.12% and ≥ 0.99, respectively.

### 2.4. Functional Gene Amplicon Sequencing and Processing

The bacterial *nirK*, *nirS,* and *nosZ* genes were amplified using the primers presented in Table S1. Prior to PCR, the 5′ end of the forward primers were modified by adding the forward Illumina Nextera adapter, a two base pair ‘‘linker’’ sequence and a unique 7-bp barcode sequence. The 5′ end of the reverse primers were modified by adding the appropriate reverse Illumina Nextera adapter and linker sequence. The PCR products were purified via a PCR Purification Kit (Axygen Scientific Inc., Union City, California, USA) according to the manufacturer’s instructions, and diluted to a concentration of 10 ng mL^−1^. The diluted PCR products were then paired-end and sequenced on the Illumina HiSeq sequencer at Genepioneer Biotechnology Co., Ltd. (Nanjing, China). 

Raw reads of the paired-end sequencing were merged with the software of Fast Length Adjustment of Short Reads (FLASH), and the sequences with low quality were distinguished using the software of Quantitative Insights Into Microbial Ecology (QIIME) [44]. The chimeric composite sequences were detected and screened using Usearch [45,46]. Thereafter, the tool of FrameBot on the Ribosomal Database Project (RDP) FunGene Pipeline (http://fungene.cme.msu.edu/FunGenePipeline) was used to screen the remaining high-quality sequences for frame shifts. Afterwards, bacterial *nirK*, *nirS,* and *nosZ* sequences were subjected to similarity search according to the GenBank non-redundant nucleotide database (nt) on NCBI with the blastn algorithm (http://www.ncbi.nlm.nih.gov). Finally, Operational Taxonomic Units (OTUs) for each sample were obtained by clustering sequences using the program of Cluster Database at High Identity with Tolerance (CD-HIT-EST) based on 97% minimum sequence identity [47]. The Shannon-Weiner index and Simpson index of *nirK*, *nirS,* and *nosZ* denitrifying genes in each sample were calculated using R 3.3.2 with the “vegan” packages. All the raw sequences obtained in this study have been deposited in GenBank under accession no. PRJNA540076.

### 2.5. Network Analysis

According to the descriptions by Zhou et al. [35] and Deng et al. [36], network analysis was conducted on each of the *nirK*, *nirS,* and *nosZ* denitrifying bacterial communities using the molecular ecological network analyses pipeline (http://ieg2.ou.edu/MENA/main.cgi). Before the analysis, the OTU table was square-root transformed, and only the OTUs that occurred in more than half of the samples were included in the network analysis, then a cutoff value (similarity threshold, st.) for the similarity matrix was generated automatically. A link between the pair of OTUs was constructed only if the correlation between their abundance was larger than the threshold. After network construction, the network properties were calculated by performing the “global network properties”, “individual nodes’ centrality”, and “module separation and modularity” in the pipeline. A module is a group of nodes connected more intensively to each other than to other nodes outside the group, and the modularity is used to evaluate how well a network is divided into modules. The clustering coefficient means the tendency of neighbors of a node to connect, and the shortest path length between the connections of two nodes is represented by the geodesic distance (path distance). To visualize the network explicitly, three files were generated from the “output of Cytoscape visualization” process. The three files were then loaded onto the Cytoscape for visualization of the network according to the instruction on the website (http://manual.cytoscape.org/en/stable/). 

To evaluate the topological role of each node in a network, a scatter chart was drawn using the values *Zi* and *Pi* generated from network construction. The *Zi* value represents the connectivity of the node within a module, and the *Pi* value measures the degree of a node connected with other modules [48]. Based on the simplified criteria, all species can be assigned into four groups: peripherals (*Zi* < 2.5 and *Pi* < 0.62) which links to the nodes within their own modules, connectors (*Pi* > 0.62), and module hubs (*Zi* > 2.5) that are connected to many nodes in their individual modules or to others, and network hubs (*Zi* > 2.5 and *Pi* > 0.62) served as super generalists that act as both module hub and connector [48]. For the ecological significance, the connectors and module hubs act as keystone species that play a critical role in determining the whole network structure and function. 

### 2.6. Statistical Analysis

Independent sample *t*-test was used to detect the differences of soil physiochemical properties and *nirK*, *nirS,* and *nosZ* genes abundance between the AM and PM plantations. The difference of the abundance or diversity among the three functional genes within AM or PM plantations was examined using one-way analysis of variance (ANOVA) with Turkey’s multiple comparison. Spearman correlation analysis was used to test the correlations of denitrifying bacterial community abundance and diversity with soil properties. To compare the patterns of the structure for *nirK*, *nirS,* and *nosZ* denitrifying bacteria between the two plantations, and to detect the determinants of the structure patterns, the canonical correspondence analysis (CCA) or redundancy analysis (RDA) was performed with the OTU table and the matrix of soil physicochemical properties. According to ter Braak and Smilauer [49], the choice of CCA and RDA could be judged from the results of detrended correspondence analysis (DCA). If the first axis of the lengths of gradient is greater than 4.0, the CCA will be better. Otherwise, the RDA will be better. Here, we found that the *nirS* and *nosZ* data were best suited for the unimodal model (CCA), while the *nirK* data was proper for the linear model (RDA). Prior to the analyses, the normality (Kolmogorov–Smirnov test) of each variable was tested, and the variable was log-transformed if necessary. In addition, the properties that showed strong correlations (r^2^ > 0.7) were excluded in the CCA and RDA to avoid the collinearity, and the significance of the remaining properties in affecting the denitrifying bacterial structure was identified. The CCA and RDA were performed using R 3.3.2 with the “vegan” packages. 

## 3. Results

### 3.1. Soil Physicochemical Properties

The AM plantations had significantly higher soil MBN, MBC, SOM, NH_4_^+^, SWC, and DNA content than the PM plantations (*p* < 0.05, Table 1). The soil DOC and NO_3_^−^ content in AM plantations were 11% and 21% greater than those in PM plantations, respectively. Soil pH had an average value of 3.42 in AM and 3.55 in PM, indicating strong acidification of the soils (Table 1).

### 3.2. Abundance, Diversity, and Composition of Denitrifying Bacterial Communities 

The average abundance of *nirK* and *nosZ* genes in AM plantations was 45% and 73% higher than the PM plantations, respectively (*p* > 0.05, Table 2). However, the *nirS* gene was more abundant in the PM plantations (1.83 × 10^6^ ± 6.04 × 10^5^) compared to AM plantations (9.83 × 10^6^ ± 3.95 × 10^6^) (*p* > 0.05, Table 2). The *nosZ* community had higher α-diversity than the *nirK* and *nirS* communities in both types of plantations, as indicated by the highest values of both Shannon-Winer and Simpson indices for *nosZ* genes, compared to those for the *nirK* and *nirS* genes (Table 2). The *nirS* and *nosZ* communities showed greater α-diversity in the PM than those in the AM plantations according to the Shannon-Winer estimator (*p* < 0.05, Table 2), whereas the α-diversity of *nirK* community showed no significant difference between the two types of plantations. 

**Figure 1:**
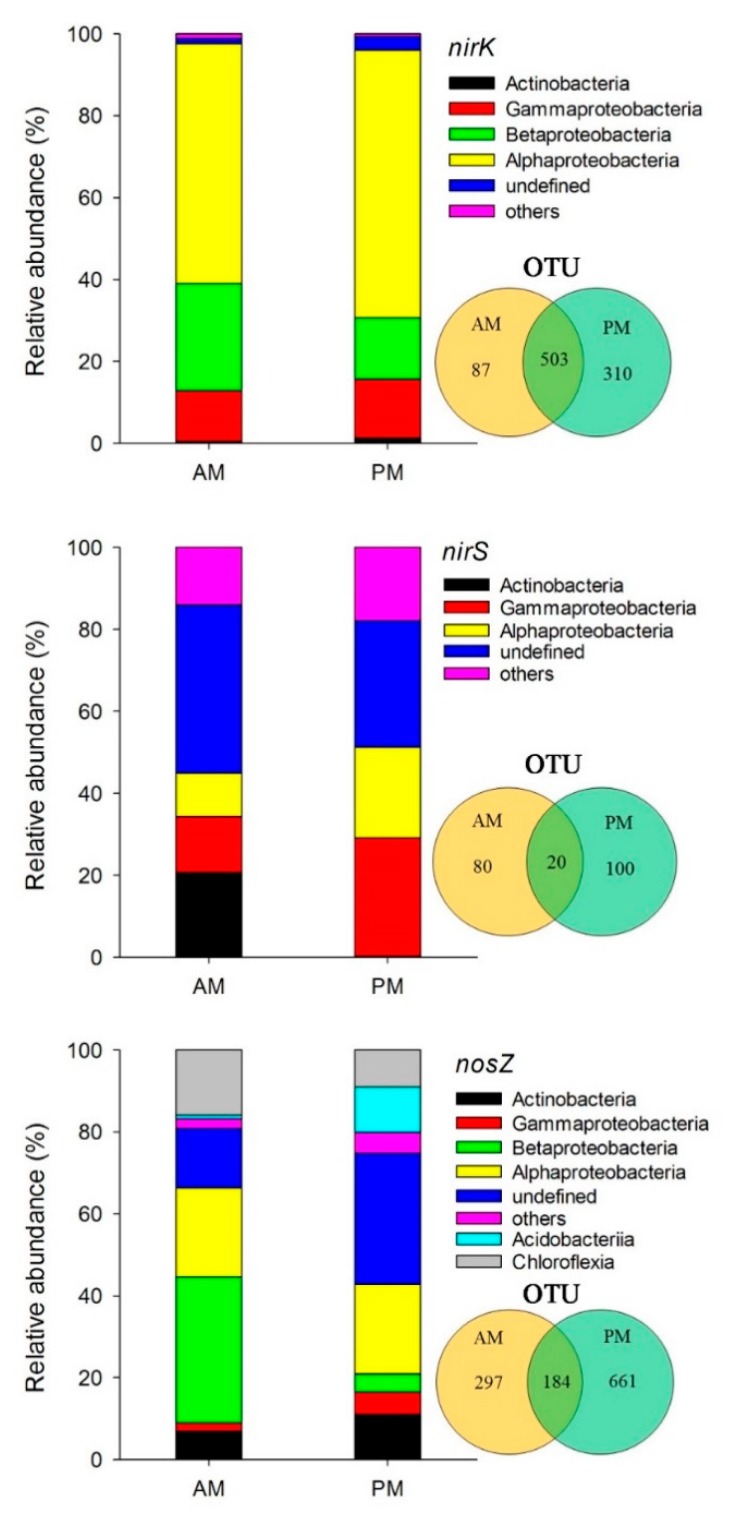
Composition of the *nirK*, *nirS*, and *nosZ* genes containing denitrifying bacterial communities in the *Acacia mangium* (AM) and *Pinus massoniana* (PM) plantations. The pie charts show the specific and shared Operational Taxonomic Units (OTUs) in the two types of plantations.

The total high-quality sequences were for *nirK* gene, for *nirS* gene, and for *nosZ* gene. According to 97% sequence similarity, a total of 900, 200, and 1142 OTUs were obtained for *nirK*, *nirS*, and *nosZ* genes, respectively. Moreover, the number of OTUs for all the three genes was higher in AM plantations than in PM plantations (Figure 1). The OTU numbers shared by AM and PM plantations were 503 for *nirK*, 20 for *nirS*, and 184 for *nosZ*, occupying 56%, 10%, and 16% of the total *nirK*, *nirS*, and *nosZ* OTUs, respectively (Figure 1). 

Regarding the community composition, four main phyla of the *nirK* denitrifying bacteria community were identified, which accounted for 96% and 98% of the total sequences in AM and PM plantations, respectively. Alphaproteobacteria was the most abundant phylum in both types of plantations (occupying 59–65% of the total *nirK* sequences), followed by betaproteobacteria (occupying 15–26% of the total *nirK* sequences), gammaproteobacteria (occupying 12–14% of the total *nirK* sequences), and actinobacteria (occupying 0.5–1% of the total *nirK* sequences). The phyla that had a relative abundance lower than 1% were assigned as others (Figure 1). Six phyla of the *nirS* denitrifiers were detected, with specific predominant phyla in each type of plantations. In AM plantations, the gammaproteobacteria, alphaproteobacteria, and negativicutes were the predominant phyla for *nirS* community that accounted for 68% of the total sequences, and in PM plantations, the predominant phyla for *nirS* community were actinobacteria, gammaproteobacteria, alphaproteobacteria, and thermoleophilia, accounting for 55% of the total *nirS* sequences from PM plantations. As much as 31–41% of the *nirS* sequences could not be assigned to any existing phyla, which was categorized as the “undefined” group (Figure 1). For the *nosZ* denitrifying bacterial community, the phyla alphaproteobacteria, actinobacteria, and acidobacteria dominated in the AM plantations, which accounted for 44% of the total *nosZ* sequences in AM plantations. The betaproteobacteria, alphaproteobacteria, and chloroflexia, occupying 74% of the total *nosZ* sequences from PM plantations, dominated in PM plantations. Moreover, the undefined group of *nosZ* community was abundant in both types of plantations, with a relative abundance of 31% in AM and 14% in PM. 

### 3.3. Relating the Denitrifying Bacterial Communities to Soil Properties

**Figure 2:**
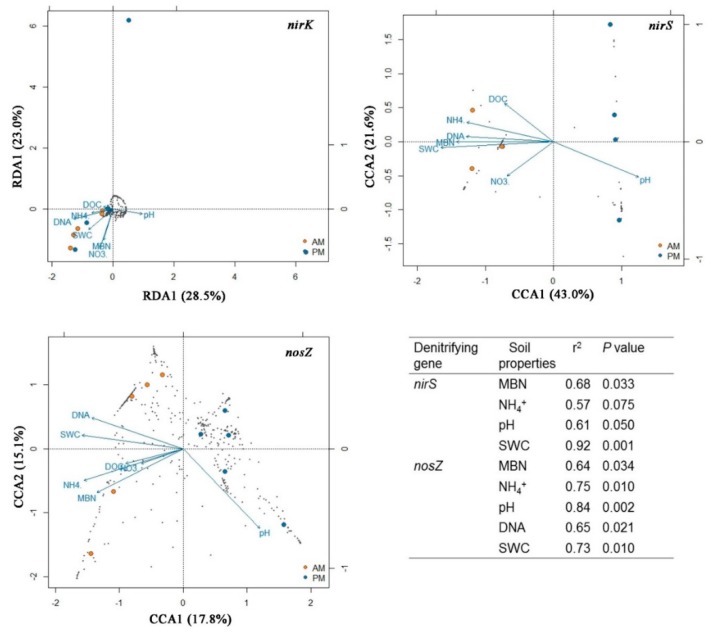
Redundancy analysis (RDA) and canonical correspondence analysis (CCA) illustrating the specific structure of the denitrifying bacterial communities and the associated factors in the *Acacia mangium* (AM) and *Pinus massoniana* (PM) plantations. Soil properties that are significantly correlated to the denitrifying bacterial community structure are shown in the table at the lower right corner.

Spearman correlations revealed that the MBC, MBN, SOM, NH_4_^+^, and SWC contents were positively correlated to *nosZ* gene abundance while being negatively related to the α-diversity of *nirS* and *nosZ* genes (*p* < 0.05, Table S2). Additionally, soil pH was negatively associated with *nosZ* gene abundance while it was positively related to α-diversity of *nosZ* gene (*p* < 0.05, Table S2). RDA results showed that the *nirK* community structure could not be explained by the differences of measured soil properties between the two types of plantations (Figure 2). The CCA profiles demonstrated that the first two CCA dimensions could explain 64% of the variation in *nirS* community structure. In contrast, only 47% of the variation in *nosZ* community structure could be explained by the first three CCA dimensions (Figure 2). The structures of both *nirS* and *nosZ* denitrifying bacteria communities were strongly correlated to MBN (*p* < 0.05), NH_4_^+^ (*p* < 0.1), pH (*p* ≤ 0.05), and SWC (*p* ≤ 0.01) (Figure 2). 

### 3.4. Denitrifying Bacteria Community Networks and the Keystone Species

**Figure 3:**
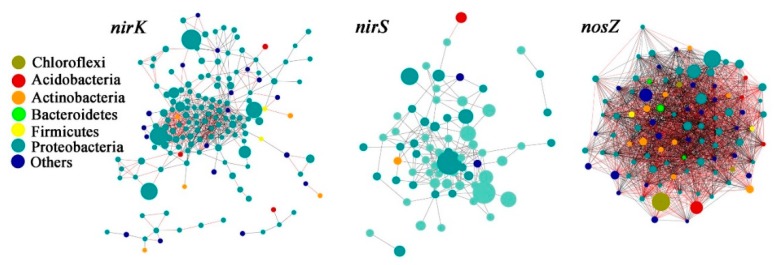
Species co-occurrence networks for the *nirK*, *nirS*, and *nosZ* gene containing bacterial communities. All OTUs from both the *Acacia mangium* (AM) and *Pinus massoniana* (PM) plantations were included in the networks. Seven bacterial phyla were represented with different colors, and the node size is proportional to the square root of the abundance of the corresponding OTU. The positive and negative correlations between the two nodes were indicated with black and red edges, respectively.

The total number of nodes within the *nirK* network (164) was greater than the *nirS* (70) and *nosZ* (95) networks, whereas the number of nodes links was the most in *nosZ* network (2079), followed by *nirK* network (634) and *nirS* network (169) (Table S3, Figure 3). The average path length (GD) was 3.67 for the *nirK* network, 3.00 for *nirS*, and 1.54 for *nosZ* networks. The *nirS* network was less modular than *nirK* network, with a lower modularity and less modules (Table S3). For the *nosZ* network, however, the modularity value was only 0.1, suggesting that the *nosZ* network was not modular. This was consistent with the results showing 15 modules in the constructed *nirK* network, while only showing two modules in the *nosZ* network (Table S3). 

**Figure 4:**
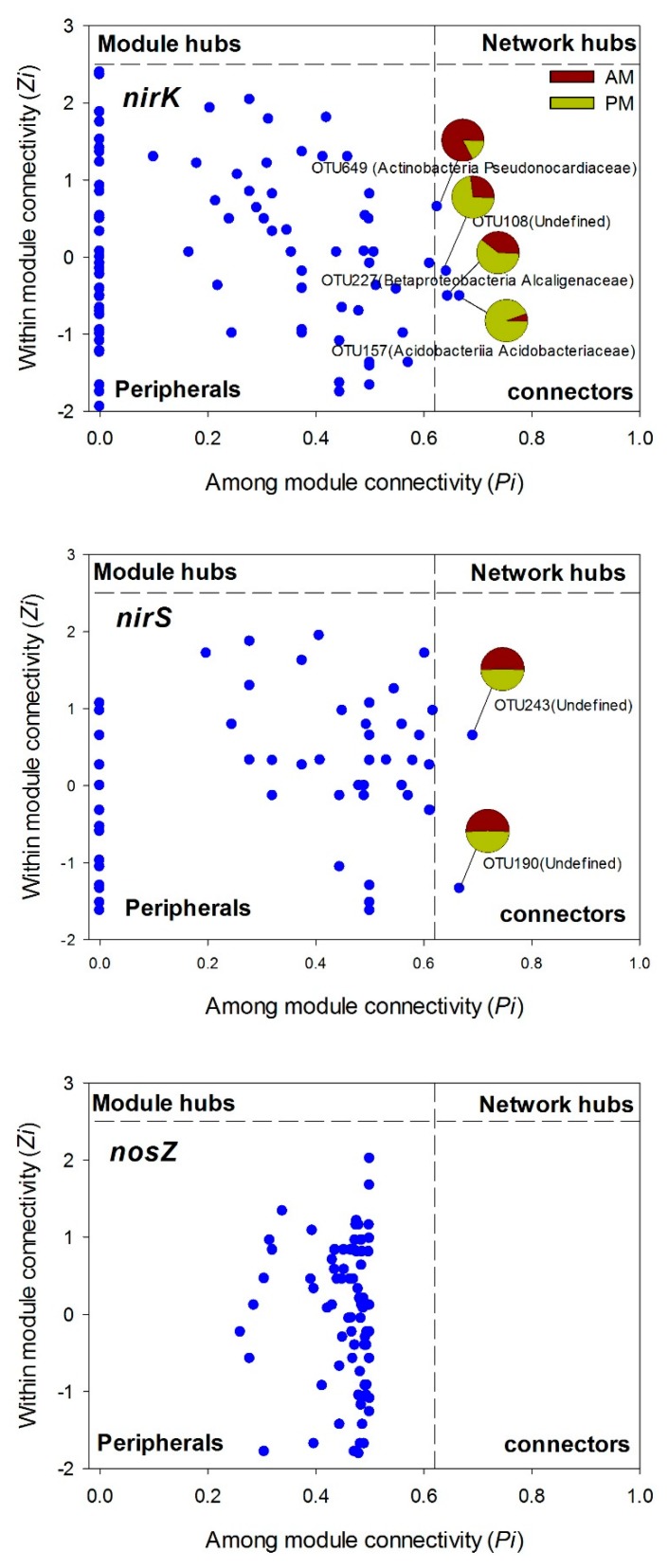
*Zi*-*Pi* plots showing the topological distribution of OTUs in the *nirK* and *nosZ* gene containing bacterial networks. The keystone species (module hubs and connectors) are marked by OTU number, and the proportion of the total number of each keystone species detected in the *Acacia mangium* (AM) and *Pinus massoniana* (PM) plantations are shown in the pie charts (i.e., the number of OTUs in AM or PM plantations divided the total OTU numbers detected in both types of plantations).

According to the topographical roles of each node (OTU in this study) in the network, the nodes categorized into the generalists (module hubs and connectors) are defined as keystone species, as these nodes are highly connected to many nodes in their own modules or other modules (Figure 4). As a result, only four nodes (2% of the total nodes) assigned to actinobacteria *pseudonocardiaceae* (OTU649), betaproteobacteria *alcaligenaceae* (OTU227), acidobacteria *acidobacteriaceae* (OTU157), and undefined bacteria (OTU108) were identified as keystone species in the *nirK* network. PM and AM plantations contained different proportions of the total number of these keystone species. For instance, 73% of the total number of OTU108, 94% of the total number of OTU157, and 61% of the total number of OTU227 were presented in the AM plantations; while 83% of the total number of OUT649 occurred in the PM plantations (Figure 4). Two undefined bacterial nodes (OTU190 and OTU243) were categorized into connectors in the *nirS* network, with 50% of the total number of the two OTUs presented in AM plantations and the other 50% in PM plantations (Figure 4). In the *nosZ* network, however, all the nodes were grouped into specialists (peripherals), and no keystone species were detected.

## 4. Discussion

This study characterized and explained the abundance, α-diversity, structure, and species co-occurrence networks of soil denitrifying bacteria communities in *Acacia mangium* (AM) and *Pinus massoniana* (PM) monoculture plantations in a subtropical region of Southern China. We aimed to provide a comprehensive understanding of the responses of soil denitrifier communities to the effects of these two tree species. We found that the abundance of *nirK* and *nosZ* genes harboring denitrifiers in AM plantations was 80–270% higher than those in the PM plantations (Table 2). Denitrifiers are mostly heterotrophs and anaerobes, and the abundance of soil denitrifying genes is generally positively related to soil moisture and organic substrates content [25,28,50]. Thus, the higher abundance of *nirK* and *nosZ* genes in AM plantations probably resulted from the higher soil C and N content, as well as higher SWC in the AM plantations (Table 1). However, the *nirS* gene was five times more abundant in the PM plantations compared to AM plantations (Table 2). Although a recent study suggested that the *nirS* denitrifying bacteria was substantially affected by soil pH in both natural and re-vegetated subtropical forests in Southern China [51], the soil pH value was similar in the PM and AM plantations. Thus, the difference in *nirS* gene abundance between PM and AM plantations might be caused by other important factors, such as soil manganese, O_2_, and texture [21]. Unexpectedly, only the *nosZ* gene abundance showed significant correlations with soil physicochemical properties (Table S2). This, to some extent, reflected a greater sensitivity of *nosZ* denitrifiers to environmental changes caused by the two types of plantations than the *nirK* and *nirS* denitrifiers. Previous research suggested that the energy produced during denitrification decreased gradually with the sequential reduction of substrates [52]. Thus, the *nosZ* denitrifier may be more sensitive to changes in exogenous energy resources from available soil nutrients, as this denitrifying community conducts the last step of denitrification [21]. Concluding, the higher soil C and N content, and higher soil moisture in the AM plantations, supported a higher abundance of *nirK* and *nosZ* denitrifying communities, with the potential for serious consequences, such as higher soil denitrification rate and greater gaseous N losses in the AM plantations compared to the PM plantations. 

The *nirS* and *nosZ* denitrifying communities had significantly higher α-diversity (Shannon-Weiner index) in the PM plantations compared to the AM plantations (Table 2). Moreover, the OTU richness of the three denitrifier communities was higher in the PM plantations (Figure 1). Obviously, the diversity and species richness of denitrifiers showed a negative correlation with soil nutrients (i.e., MBC, MBN, SOM, and NH_4_^+^) and water content (Table S2). These results may be due to the fact that a large species pool could enable the microbial community to resist unfavorable environmental conditions [53] as the infertile soils under PM plantations in this study. It is also possible that a microbial community with high diversity might be required to adapt to the low quality and quantity of soil organic matter in the PM plantations [54]. These speculations could be further supported by the positive correlations of *nirS* and *nosZ* denitrifiers structure with higher soil MBN, NH_4_^+^, and SWC in the AM plantations (Figure 2). Specifically, MBN could serve as a potential N substrate after being released by dying microorganisms [55]. NH_4_^+^ content generally showed an indirect effect on denitrifiers through affecting the ammonium oxidization process, as the products of ammonium oxidization (i.e., NO_2_^−^ and NO_3_^−^) are fundamental substrates for denitrifiers. 

It is worth noting that the structure of the *nirK* denitrifier could not be separated by the differences in the soil properties between the AM and PM plantations (Figure 2), consisting of a large overlap of community composition between the two types of plantations (Figure 1). One reason might be that the variation in *nirK* composition between AM and PM plantations was overwhelmed by the variation among the samples within PM plantations (Figure 2). Thus, more soil samples need to be collected to generate a more precise *nirK* composition in the future studies. It is also possible that the *nirK* community structure in the two types of plantations is determined by other unmeasured soil properties, such as phosphorus, potassium, calcium, and copper [21,56]. In order to explore the species connections in *nirK* community structure and their driving mechanisms, we conducted a species co-occurrence network analysis. Results showed that the modularity value of *nirK* network was 0.5, which was similar to the values from the most modular networks [36], reflecting a modular property of the *nirK* network. Moreover, the average path length (GD, the efficiency of information or mass transport on a network) for the *nirK* network was higher than those of the *nirS* and *nosZ* networks (Table S3), indicating that the *nirK* community displayed a more efficient small-world behavior [36]. Thus, instead of separation with plantation types, the *nirK* denitrifying community was modularly organized under the AM and PM plantations. The keystone species in the network might play a predominant role in determining the structure of the overall community, as these species are closely connected to the OTUs within or outside of the modules [35]. Additionally, most of the keystone species from *nirK* network occurred in PM plantations compared to AM plantations (Figure 4), indicating a better organized or a better operational *nirK* community in the PM plantations versus the AM plantations [37]. It is possible that in PM plantations, the interspecies corporation in *nirK* community is more intensive, aiming to resist the nutrient depletion. However, the way in which different species incorporate with each other needs further study. In the present study, the *nirK* community structure showed no significant correlations with soil properties (Figure 2), suggesting that the species interconnection in the *nirK* network might not be driven by an environmental filter process. The stochastic or other deterministic mechanisms, including functional association, physical contact, or phylogenetic clustering of closely related species may play a key role in governing *nirK* denitrifier community structure in the AM and PM plantations [35]. In summary, the *nirS* and *nosZ* denitrifying bacterial communities showed divergent structure between the AM and PM plantations, which was mainly related to the differences in soil organic and inorganic N, pH, and water content between the two types of plantations. The structure of *nirK* denitrifying bacteria, however, could not be separated by plantation type, exhibiting a modular species co-occurrence network as a whole.

## 5. Conclusions

This study compared the abundance, diversity, and structure of soil denitrifying bacterial communities between *Acacia mangium* and *Pinus massoniana* plantations in a subtropical region of South China by quantifying and sequencing the functional genes *nirK*, *nirS*, and *nosZ*. The two types of plantations had distinct structure of *nirS* and *nosZ* denitrifying bacteria, which are mainly associated with the different soil properties (i.e., MBN, NH_4_^+^, pH, and SWC) between the two types of plantations. However, the plantation type did not separate the structure of the *nirK* denitrifying community, resulting in a large species overlap between the AM and PM plantations. The *nirK* community showed a higher resistance to environmental changes, mainly due to a well-organized community network with greater modularity and more keystone species. These results demonstrate that the diversity and structure of *nirS* and *nosZ*, but not *nirK*, communities are sensitive to soil environmental changes caused by *Acacia mangium* and *Pinus massoniana* monoculture plantations. Thus, investigations of the effects of *nirS* and *nosZ* denitrifiers changes with forest conversion to plantations on soil N cycling warrant further study. In addition, the greater abundance of denitrifying genes and higher soil N availability in AM can enhance N losses and make the N cycling more “open”. We suggest that planting *Pinus massoniana* trees is better than *Acacia mangium* at the initial stage of forest restoration in terms of soil N accumulation. 

## Figures and Tables

**Table 1:** The average soil physicochemical properties and total DNA (μg g^−1^) content in the *Acacia mangium* (AM) and *Pinus massoniana* (PM) plantations. The average value was calculated from five replicates in each type of plantation, and the standard error is shown in brackets. Different lowercases mean the significant difference between the AM and PM plantations at *p* < 0.05.

Sites	SOM	DOC	MBC	MBN	NH_4_^+^	NO_3_^−^	SWC	pH	DNA
AM	111.82a (6.59)	219.81a (17.84)	367.42a (42.22)	71.23a (9.08)	12.17a (3.41)	13.75a (1.71)	35.37a (1.07)	3.42a (0.03)	11.95a (1.48)
PM	70.65b (5.65)	195.63a (9.73)	213.15b (23.01)	39.61b (4.29)	2.22b (0.81)	10.83a (1.53)	23.15b (1.21)	3.55a (0.03)	5.47b (1.79)

Abbreviations: SOM, soil organic matter (g kg^−1^); DOC, dissolved organic carbon (mg C kg^−1^); MBC, microbial biomass carbon (mg C kg^−1^); MBN, microbial biomass nitrogen (mg N kg^−1^); SWC, soil water content (%).

**Table 2:** The average abundance and *α*-diversity of *nirK*, *nirS*, and *nosZ* gene harboring denitrifying bacterial communities in the *Acacia mangium* (AM) and *Pinus massoniana* (PM) plantations. The *α*-diversity is represented by Shannon-Weiner and Simpson indices. The average value was calculated from five replicates in each type of plantation, and the standard error is shown in brackets. Different lowercases mean the significant difference between AM and PM plantations, and different capitals indicate the significant difference among the three genes within one type of plantations. The significance is set at *p* < 0.05.

Sites	Abundance			Shannon-Weiner Index			Simpson Index		
	*nirK*	*nirS*	*nosZ*	*nirK*	*nirS*	*nosZ*	*nirK*	*nirS*	*nosZ*
**AM**	6.28 × 10^5^aA (2.12 × 10^5^)	1.83 × 10^6^aB (6.04 × 10^5^)	2.01 × 10^7^aC (2.76 × 10^6^)	2.33aAB (0.32)	1.57bA (0.14)	2.83bB (0.27)	0.75aA (0.08)	0.65aA (0.04)	0.81aA (0.07)
**PM**	3.48 × 10^5^aA (1.70 × 10^5^)	9.83 × 10^6^aB (3.95 × 10^6^)	5.40 × 10^6^aB (2.31 × 10^6^)	2.51aA (0.30)	2.40aA (0.20)	3.78aB (0.11)	0.78aA (0.07)	0.87aAB (0.03)	0.95aB (0.01)

## Supplementary Materials

The following are available online at https://www.mdpi.com/2076-2607/7/9/361/s1, Table S1: Primers and thermal profiles used for the quantification of denitrifying functional genes, Table S2: Spearman’s correlation between the abundance and α diversity of denitrifying bacterial community and soil physicochemical properties, Table S3: Main properties of the species co-occurrence networks for *nirK*, *nirS* and *nosZ* denitrifying bacterial communities.
