# Supplementary material for: The Structure and Species Co-Occurrence Networks of Soil Denitrifying Bacterial Communities Differ Between A Coniferous and A Broadleaved Forests
Source: Microorganisms. 2019 Sep 18;7(9):361. doi: 10.3390/microorganisms7090361 (PMC6780695; doi:10.3390/microorganisms7090361)
Supplement: Supplementary file 1 [file microorganisms-07-00361-s001.pdf]

The Structure and Species Co-Occurrence Networks of Soil Denitrifying Bacterial Communities Differ Between A Coniferous and A Broadleaved Forests

Jie Chen [1,*], Jiajia Li [2,*], Weijun Shen [3], Han Xu [1], Yide Li [1], and Tushou Luo [1]

[1] Research Institute of Tropical Forestry, Chinese Academy of Forestry, Longdong, Guangzhou 510520, China; ywfj@163.com (H.X.); liyide@caf.ac.cn (Y.L.); luots@126.com (T.L.)
[2] College of Tourism and Planning, Pingdingshan University, Pingdingshan 467000, China;
[3] Key Laboratory of Vegetation Restoration and Management of Degraded Ecosystems, South China Botanical Garden, Chinese Academy of Sciences, 723 Xingke Rd. Tianhe District, Guangzhou 510650, China; shenweij@scbg.ac.cn
* Correspondence: chenjiecaf@hotmail.com (J.C.); lijiajia5618@126.com (J.J.L.); Tel.: + 86 2087032619 (J.C.); +86 3752077263 (J.J.L.)

Table S1. Primers and thermal profiles used for the quantification of denitrifying functional genes.

Target gene	Primer name	Target gene length	Thermal profile	Reference
nirK	F560-589/ R906-935	376bp	30 s /95 °C - 40 cycles (5 s/ 95 °C - 34 s / 65 °C) - 1 min / 72 °C	Chenier et al., (2003)
nirS	*nirS* 1F/*nirS* 3R	256bp	30 s /95 °C - 40 cycles (5 s/ 95 °C - 34 s / 65 °C) - 1 min / 72 °C	Braker et al. (1998)<br>Levy-Booth and Winder (2010)
nosZ	*nosZ* 2F/*nosZ* 2R	267bp	30 s /95 °C - 40 cycles (5 s/ 95 °C - 34 s / 65 °C) - 1 min / 72 °C	Henry et al. (2006)

Table S2 Spearman's correlation (ρ) between the abundance and α diversity of denitrifying bacterial community and soil physicochemical properties in the *Acacia mangium* and *Pinus massoniana* plantations (n = 10).

Functional gene index	SOM	DOC	MBC	MBN	NH_4^+	NO_3^-	SWC	pH
NirK abundance	0.43	-0.09	0.42	0.13	0.33	-0.39	0.41	-0.62
NirS abundance	-0.25	-0.10	-0.46	-0.61	-0.22	-0.47	-0.32	0.12
NosZ abundance	0.86**	0.19	0.75*	0.66*	0.64*	-0.02	0.83**	-0.89**
Shannon index of *nirK* gene	-0.14	-0.20	0.31	0.25	-0.02	0.21	0.02	0.24
Shannon index of *nirS* gene	-0.78**	-0.27	-0.64*	-0.59	-0.65*	0.24	-0.69*	0.55
Shannon index of *nosZ* gene	-0.71*	-0.12	-0.75*	-0.84**	-0.49	-0.37	-0.81**	0.72*
Simpson index of *nirK* gene	-0.04	-0.19	0.37	0.24	0.06	0.24	0.12	0.07
Simpson index of *nirS* gene	-0.83**	-0.29	-0.75*	-0.71*	-0.67*	0.09	-0.79**	0.61
Simpson index of *nosZ* gene	-0.75*	-0.18	-0.70*	-0.76*	-0.52	-0.09	-0.76*	0.75*

Abbreviations: SOM, soil organic matter (g kg^{-1}); DOC, dissolved organic carbon (mg C kg^{-1}); MBC, microbial biomass carbon (mg C kg^{-1}); MBN, microbial biomass nitrogen (mg N kg^{-1}); SWC, soil water content (%). The stars represent statistically significant, with $**p \leq 0.01$; $*p \leq 0.05$.

Table S3 Main properties of the *nirK, nirS* and *nosZ* gene containing denitrifying bacterial networks. All OTUs from the *Acacia mangium* and *Pinus massoniana* plantations were included in the network. Both the properties from Empirical networks and Random networks were presented.

Parameters	*nirK*	*nirS*	*nosZ*
Empirical networks			
Similarity threshold (S_t)	0.93	0.87	0.42
Total nodes	164	70	95
Total links	634	169	2079
Number of module	15	6	2
R^2 of power-law	0.59	0.55	0.1
Average connectivity (avgK)	7.73	4.83	43.77
Average cluster coefficient (avgCC)	0.35	0.02	0.56
Average path distance (GD)	3.67	3.00	1.54
Modularity	0.50	0.41	0.1
Random networks			
Average cluster coefficient (avgCC)	0.115±0.011	0.062±0.017	0.538±0.002
Average path distance (GD)	2.831±0.038	2.888±0.064	1.535±0.000
Modularity	0.279±0.006	0.363±0.014	0.062±0.006

References

Braker G, Fesefeldt A, Witzel KP (1998) Development of PCR primer systems for amplification of nitrite reductase genes (*nirK* and *nirS*) to detect denitrifying bacteria in environmental samples. Applied and Environmental Microbiology 64: 3769-3775

Chenier MR, Beaumier D, Roy R, Driscoll BT, Lawrence JR, Greer CW (2003) Impact of seasonal variations and nutrient inputs on nitrogen cycling and degradation of hexadecane by replicated river biofilms. Applied and Environmental Microbiology 69: 5170-5177

Henry S, Bru D, Stres B, Hallet S, Philippot L (2006) Quantitative detection of the nosZ gene, encoding nitrous oxide reductase, and comparison of the

abundances of 16S rRNA, *narG*, *nirK*, and *nosZ* genes in soils. Applied and Environmental Microbiology 72: 5181-5189

Levy-Booth DJ, Winder RS (2010) Quantification of Nitrogen Reductase and Nitrite Reductase Genes in Soil of Thinned and Clear-Cut Douglas-Fir Stands by Using Real-Time PCR. Applied and Environmental Microbiology 76: 7116-7125.
